# Association Between Individual Animal Traits, Competitive Success and Drinking Behavior in Dairy Cows After Milking

**DOI:** 10.3390/ani15040534

**Published:** 2025-02-13

**Authors:** Franziska Katharina Burkhardt, Rieke Wahlen, Jason Jeremia Hayer, Julia Steinhoff-Wagner

**Affiliations:** 1 TUM School of Life Sciences, Technical University of Munich, Liesel-Beckmann-Str. 2/II, 85354 Freising-Weihenstephan, Germany; 2 Educational and Research Centre for Animal Husbandry Hofgut Neumuehle, Neumühle 1, 67728 Muenchweiler an der Alsenz, Germany; 3 HEF World Agricultural Systems Center, Technical University of Munich, 85354 Freising-Weihenstephan, Germany

**Keywords:** cattle, water uptake, social competition, welfare indicator

## Abstract

After milking, dairy cows seek the nearest water trough, but access depends on competitive success. This study descriptively characterized individual drinking behavior after milking in association with physiological traits and competitive success in a herd of 42 lactating Brown Swiss cows held in a free-range barn. Cows were milked and fed twice a day, and the drinking behavior was video recorded after leaving the milking parlor during 22 milkings in July 2022. Water consumption and 33 behavioral parameters were recorded. Competitive success was defined by an index calculation. Milk production traits correlated positively with the number of trough visits, the total duration of drinking, the duration of water intake and drinking breaks, and the water volume consumed. High-yielding dairy cows exhibited twice as many agonistic interactions as low-yielding ones, suggesting that performance-based grouping increases aggressive interactions. Further, subtle dominance interactions, such as “staring”, comprise a major part of confrontations at the trough. Cows with low competitive success more frequently pursued other activities before they drank and were more frequently interrupted by agonistic interactions. Considering body and performance traits in association with individual drinking patterns in evaluating on-farm water management seemed valuable, therefore, a review of current recommendations for water supply is suggested.

## Simple Summary

Accessibility to water is essential for dairy cows’ health and welfare but may be limited by competitive success. This study investigated dairy cows’ behavior at the trough after milking, considering specific body and performance traits and their influence on competitive success. Cows that produced more milk tended to visit the trough more often, drink for longer, and consume more water. These high-producing cows also competed more aggressively, often using physical or non-physical behaviors like staring to dominate. In contrast, less dominant cows avoided conflict, waiting until troughs were free, but their drinking was often interrupted. These findings suggest that grouping cows based on their milk production can increase competition, so farms may need more water troughs in high-producing groups to ensure equal access. Better understanding of how cows drink and interact may help farmers improve water access, reduce stress, and support the health and productivity of their animals, highlighting the need to reassess water management practices on dairy farms.

## 1. Introduction

Accessibility to water is essential for dairy cows to meet their nutritional, behavioral, and thermoregulatory needs. As described by Burkhardt et al. [1], the process of water intake demands a certain amount of time, as cows rather taste the water before actually drinking. During tasting and drinking, they display various other drinking-related behavioural patterns, as “smelling”, “looking around without any recognizable sensory perception, tasting with “visible tongue”, “coughing”, or “agonistic interactions”.

Several factors influence dairy cow drinking behavior, including housing conditions, trough design [1,2,3,4], trough location [5], trough material [4], water type [6], water quality [1,7,8,9], flow rate [10], water temperature [11,12], and season [13,14,15,16]. Current studies on drinking behavior are mostly aligned to automated evaluation and, therefore, focused on the water consumed and total drinking time of a group of animals but neglect potentially relevant behavioral indicators of individual cows that cannot be monitored automatically at the current state of technology [17]. Considering a broader spectrum of specific drinking behavior parameters, thereby taking individuals into account, may help to understand the complexity of dairy cow drinking behavior and thus optimize water supply management [1,18,19].

Nevertheless, behavioral changes as potential management or animal welfare indicators must be considered in the context of other animal-specific factors such as breed, age, parity, physiological condition, and individual characteristics as those influence nutritional needs, and thus, well-being and behavior [18,19,20]. For example, water demand and consumption increase with body weight and milk yield [21,22,23]. Furthermore, Heath et al. [24] identified the “absence of prolonged thirst” as an iceberg indicator that can be used to predict the overall animal welfare reliably.

A dairy cow has an exceptionally high need for water due to the demand for milk production and thermoregulation [25]. Dairy cows drink most of their daily water requirements after milking [26]. Adequate quantity and quality water near the milking parlor is therefore especially suggested [27].

Competitive interactions at the troughs are common and can limit access to fresh water in dairy herds [1,28]. Due to its cooling effect and evaporation purposes, the number of competitive interactions near water troughs increases the higher the ambient temperatures [5,16,28,29]. Additionally, Foris et al. [30] explored the social hierarchy at drinking troughs, finding that cows with higher dominance had fewer visits but higher water intake, indicating that subtle agonistic behaviors can influence drinking patterns, suggesting that subtle agonistic behaviors are significant in understanding water intake dynamics among dairy cows. Following Hohenbrink and Meinecke-Tillmann [31], almost half of the individuals within a herd are rank-subordinate; thus, a small number of dominant cows blocking water sources possibly leads to performance losses and health issues in dairy herds. It is likely that particularly lower-ranking individuals suffer from competitive interactions at the trough [28]. Therefore, it is crucial to understand the individual’s behavior in the context of the rank order, especially after milking when water demand is high.

Current recommendations for water management often overlook subtle behavioral indicators, such as non-physical, indirect interactions and indirect reactions, such as avoidance behavior [17,32], and fail to account for individual differences based on competitive success, performance traits, and physiological needs. This creates a gap in knowledge that is critical for optimizing water access and improving animal welfare in dairy herds. Hence, to provide recommendations for water supply systems considering the number and placements of water troughs or developing management strategies to minimize competition, especially for subordinate cows, this study aimed to evaluate the effect of dairy cows’ individual drinking behavior after milking, considering performance traits and competitive success. We hypothesized that (1) the drinking behavior varies between individuals, as differences in physiological and body characteristics potentially lead to variations in the drinking behavior among individuals, (2) that cows with a high competitive success predominantly use the trough next to the milking parlor directly after milking, and (3) those cows consume more water, drink longer, show more extended periods of water intake, and start more frequently agonistic interactions than cows with less competitive success at the trough.

## 2. Material and Methods

### 2.1. Ethical Note

This study was conducted following the principles stated in Directive 2010/63/EU on the protection of animals used for scientific purposes as well as the German Welfare Act and the Animal Welfare and Farm Animal Husbandry Ordinance. We adhered to the ethical standards and data privacy agreements of TUM and the federal and institutional animal use guidelines (TUM ANM 2022/001).

Our research complies with the commonly accepted 3Rs (NSW Department of Primary Industries and Animal Research Review Panel) as

1. No intervention or treatment was administered to the animals under study.
2. The experiment was conducted within the realm of agricultural activity on a farm setting.
3. The involvement of animals in the experiment was confined solely to the capturing of video recordings.

Animals were held under permanent surveillance of a veterinarian, housed, and fed according to good animal attendance practice (Balis registration number 09 178 124 0461 from 6 July 2022) and in compliance with QM standards. No animals were sacrificed for this study, and all animals were housed for further research.

### 2.2. Experimental Facility and Animals

The study was conducted on an experimental barn of the Technical University of Munich at 450 m above sea level. The average annual rainfall and annual temperature in the area are 834 mm and 8.7 °C, respectively [33]. The experiment was performed in July 2022, corresponding to summer in the Northern Hemisphere.

**Figure 1:**
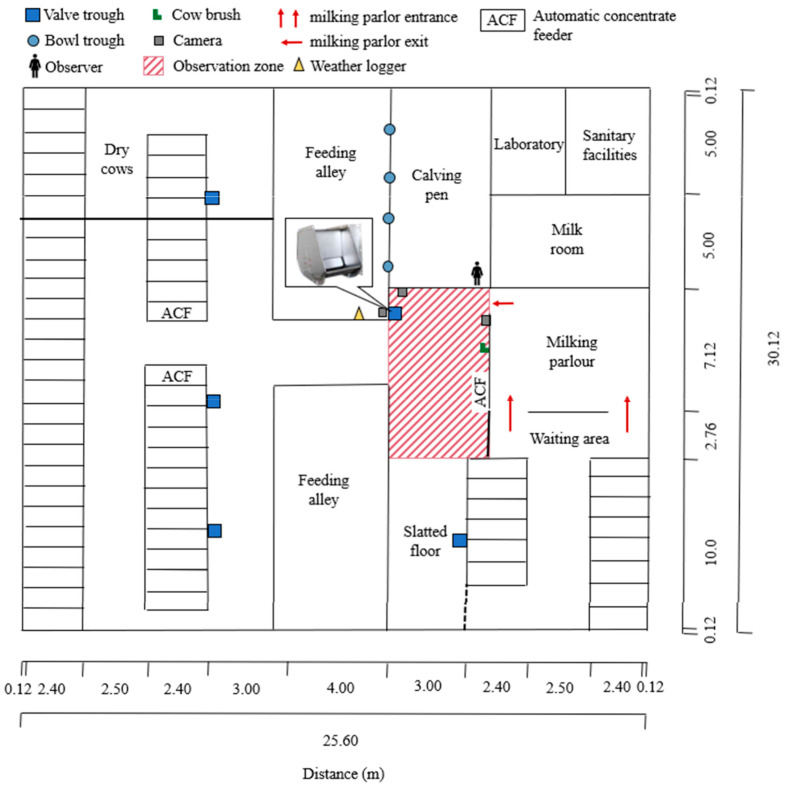
Experimental dairy cow facility used in a drinking behavior study at a trough next to the milking parlor directly after milking in *n* = 51 dairy cows. Distances are shown in meters. Farm characteristics and experimental elements are marked with a symbol and are not drawn to scale for visualization purposes. The observation zone is highlighted in red.

On the TUM experimental farm Veitshof used for this experiment, a dairy herd of 47 lactating Brown Swiss cows was continuously held in a loose-house barn (Figure 1, Table 1). The dairy barn was built for 70 cows, which led to many more feeding spaces and lying places as animals were kept. Five dry cows were excluded from the study. In total, cows had access to five valve troughs (0.36 m length, 0.32 m wide, 0.10 m depth; variable volume, 5–15 L each).

All animals were weighed and measured two weeks before the trial (14 June 2022). Individual milking performance traits were gained on 26 June (before the trial) and 31 July 2022 (after the trial) and averaged for each cow using the HI-Tier herd register (HI-Tier database, Bay.StMELF, Munich, 1999–2021) and the LKV app (LKV Rind [BY], Landeskuratorium der Erzeugerringe für tierische Veredelung in Bayern e.V. (LKV), Munich, 2022). Additionally, the non-digitalized cow register of the TUM experimental farm Veitshof served as a data basis for calculating the general data of the respective dairy cow, such as age or days in milk, and referred to the first day of observation. The day of milk sampling was also chosen as the effective date for recording general data of the respective dairy cow, such as age or days in milk.

Cows were milked in a 2 × 2 tandem herringbone milking system and fed twice a day in the morning and evening. A partially mixed ration (PMR) was offered in a centralized 29.88 m long feeding alley. Feeding alley bridge was closed only for feed provision and opened during observation period. The PMR contained corn silage [35% dry matter (DM)], grass silage (42% DM), alfalfa/grass silage (25% DM), and concentrate (35% DM). Additional concentrate was fed in three concentrate feeders (Figure 1). The drinking water entering the water supply system was well water from the local municipal utility that complied with German regulations for human drinking water quality (Supplementary Table S1) [34].

### 2.3. Experimental Procedure

The drinking behavior of the dairy cows was video-recorded daily, beginning with the first cow leaving the milking parlor until the last cow left the observation zone (3.00 × 7.20 m) around a valve trough facing the milking parlor. The study was conducted during eleven consecutive days, following a ten-day pre-trial period. Milkings comprised an average duration of 2.0 h. Video recording was conducted using three perspectives (above, aside of the trough, and above the milking parlor exit), thus, a total of 132 h of video material was analyzed. Including the on-farm observation of animal interactions, individual animal identification, plausibility checks using different perspectives, and matching the water meter data, the analysis took over 200 h. A detailed behavioral description, as provided in the current study (*n* = 33 behavioral parameters), as well as changes in the herd structure (dry off and integration after calving), did not allow for an extension of the trial period. Therefore, the observation was limited to one drinking trough. The drinking trough closest to the milking parlor was selected for this purpose as, according to various authors, water intake takes predominantly place after feeding and milking time [14,26,35]. Additionally, agonistic interactions most likely occur in situations with high stocking density, namely in areas where cows compete for resources, as the entrance and exit of the milking parlor, water, and feed bunks [28]. Therefore, it is reasonable to assume that both the drinking behavior and agonistic interactions are characterized, in particular at this trough. The trough was made of stainless steel, and the consumed volume of water was measured using a water flow meter (calibrated perfk water meter cold water meter 15 mm ½ inch, Perfk, Shenzhen, China). Meter readings were video recorded, assigned to the respective drinking episode, and subsequently analyzed. The drinking trough was cleaned twice daily at the trials beginning before the first cow left the milking parlor.

An observer was positioned in the calving pen on the opposite site of the trough during the trials to identify the individual animal visually by their collar identification number and to obtain additional information on agonistic behaviors (Figure 1). This includes (1) the initial activity each individual engages in first after milking and (2) the identification of agonistic interactions between individuals, distinguishing between the actor and receiver roles. This information was thereafter included in the data set. Close to the observational drinking trough, the only mechanical cow brush in the barn was located (Figure 1). As described by DeVries et al. [36], the presence of brushes affected dairy cows’ behavior, particularly social interactions and grooming.

Competitive success at the trough was identified by the “Index of competitive success” according to McDonald et al. [28], which is based on the “Index of displacements” of Galindo and Broom [37], adapted as (range 0–1):

$$Index\;of\;displacements=\frac{No.\;of\;times\;the\;cow\;displaced\;other\;cows}{No.\;of\;times\;the\;cow\;displaced\;another\;cow+No.\;of\;times\;the\;cow\;is\;displaced}$$

It divides the number of cow displacements by the cow by the total number of displacements the cow was involved in. The original index of Galindo and Broom [37] was modified for our study: all cows with an index of displacements < 0.4 were classified as having low competitive success and high success > 0.6. All others were grouped into “medium success”.

The trial took place for 11 days in July 2022. During a five-day pre-trial period, the cameras were installed, and the observer took position next to the milking exit to familiarize the animals with the experimental procedures. The observer became used to the documentation procedure. Additionally, cows were familiar with the presence of humans. During the pre-trial period, alternatively, the rank entering the milking parlor was tested as an indicator for competitive success but failed due to dominant cows waiting until the end before entering.

### 2.4. Analysis of Drinking Behavior

A detailed description of each drinking behavior parameter recorded, which is based on Burkhardt et al. [1,16], can be seen in Table 2. On an individual basis, the total number of “trough visits”, “total trough visits” (including cows that returned to drink), “non-drinking episodes”, “total episodes in the observation zone”, trough visits “after milking”, “after another activity”, “brushing”, “eating concentrate food”, “eating PMR”, or “leaving immediately” was counted. A drinking episode began with the cows’ heads crossing the edge of the water trough, which simultaneously was defined as the beginning of the tasting period. The tasting period was terminated either by the cow stepping away from the trough or the cow taking more than five continuous sips during the water intake. If a drinking episode ended after tasting, it was counted as “tasting only”. The entire drinking behavior was subdivided into periods of water consumption and drinking breaks. During water intake, the number of sips was additionally counted. Agonistic behaviors from the cow drinking as an antagonist were counted and clustered into “staring”, “moderate push”, “pushing aside”, and “fighting”. Likewise, during the “interruptions due to agonistic behaviors”, observed behavior of the antagonist was recorded and divided into: “ignoring the disturbance”, “interruption”, or “leaving the observation zone”. Additionally, “coughing” was counted as representing swallowing difficulties.

Four time-lapse cameras (TLC 200, Brinno, Taipei City, Taiwan) were used to record the dairy cows’ behavior with one full-color picture per second. Time-lapse cameras were recommended as particularly suitable after consulting with an IT expert, as they reduce the amount of data generated and are considered sufficient in terms of data quality for potential machine-learning-supported automated evaluation subsequent to the present analysis. These cameras were installed above, beside, and opposite the trough to capture images of the entire drinking trough area, including the cow brush, concentrate feeder, and feeding alley (Figure 1). Videos were analyzed using Behavioral Observation Research Interactive Software (BORIS, https://www.boris.unito.it/, https://doi.org/10.1111/2041-210X.12584) [38].

**Table 2:** Characterization of dairy cows’ drinking behavior used in the analysis of video-recorded behaviors.

Variable	Data Type	Unit	Description
**Behavioral episodes registered inside the observation zone**			
Trough visits ^1^	Continuous	Count	Number of episodes the cow crosses the edge of the trough at least once with its *Planum nasolabiale.* Including episodes the cow did not touch the water. Excluding visits after the cow left the observation zone at least once and returned.
Total trough visits	Continuous	Count	Total number of episodes the cow crosses the edge of the trough with its *Planum nasolabiale,* including cows that return to the trough after leaving the observation zone
Non-drinking episodes	Continuous	Count	Total number of episodes the cow did not cross the edge of the trough at least once with its *Planum nasolabiale* after leaving the milking parlor.
Total episodes in the observation zone	Continuous	Count	Total number of observed activities after milking, including visits of the cow brush, the drinking trough, the concentrate feeder, the feeding alley or leaving immediately.
Trough visits immediately after milking	Continuous	Count	Number of episodes the cow visits the drinking trough first after milking and crosses the edge of the trough with its *Planum nasolabiale.*
Trough visits after other activity	Continuous	Count	Number of episodes the cow returns to the drinking trough after another specific activity and crosses the edge of the trough with its *Planum nasolabiale.*
Brushing after milking	Continuous	Count	Number of episodes the cow visits and touches the cow brush first after milking.
Eating concentrate food after milking	Continuous	Count	Number of episodes the cow visits and touches the concentrate feeder first after milking.
Eating PMR after milking	Continuous	Count	Number of episodes the cow visits and touches the feeding alley first after milking.
**Drinking-related behaviors**			
Total duration of drinking ^1^	Continuous	s	Time elapsed from the moment a cow crosses the edge of the trough until the cow crosses the edge of the trough the last time before stepping away from the trough. The total duration of drinking includes the tasting period and is divided in periods of water intake and drinking breaks [1].
Duration of tasting period ^1^	Continuous	s	Timespan, beginning with the cow crossing the edge of the trough and ending with water intake, including short periods of water intake <5 sips with <3 s between sips and drinking breaks, or the cow leaving the trough. During this time span, cows may taste the water with a visible tongue, smelling at the water or the trough, or just looking around without any visible sensory perception [1].
Only tasting ^1^	Dichotomized	%	Begins with a cow crossing the edge of the trough and ending with the cows stepping away from the trough without water intake or including short periods of water intake <5 sips excluding water intake periods >5 sips with <3 s between sips. cows leave the trough after the tasting period [1].
Duration of water intake ^1,a^	Continuous	s	Total time the *planum nasolabiale* is under the water surface or in contact with the water surface during a total drinking event, including both a water intake of >5 sips or during a water contact with drinking breaks lasting <3 s and water intake periods of <5 sips or during a water contact with drinking breaks lasting >3 s. Sips are not necessarily represented by a visible tongue, but rather by visible neck movements at the throat when swallowing the water [1].
Water intake periods ^1^	Continuous	s	“Total number of “water intake” periods during “total drinking episode”, including both, water intake periods of >5 sips or during water contact with drinking breaks lasting 3 s.” [1].
Number of sips per drinking episode ^1,a^	Continuous	Count	Number of sips measured as counts per “drinking episode.” Total number of sips while tasting and sips per period of water intake. Sips are “a movement of the animal’s throat swallowing water, while its mouth is submerged” [2] and visible by contraction of the cheek muscle and/or marked contraction of the throat and/or clear water movement while *planum nasolabiale* is in contact with the water surface [1].
Water volume consumed	Continuous	L	Total amount of water consumed.
Water volume consumed per sip	Continuous	L	Total amount of water consumed in one up taken sip.
Duration of drinking breaks ^1^	Continuous	s	Total time during a drinking event the *planum nasolabiale* is above the water surface, i.e., not in contact with the water, including the time before, between and after water intake periods [1].
Drinking breaks ^1^	Continuous	s	Total number of “drinking breaks” during a “the total duration of drinking [1].”
Swallowing difficulties ^1^	Dichotomized	%	Coughing with throat extended, usually combined with a visible tongue [1].
**Agonistic behaviors registered during trough visits**			
Total number of agonistic behaviors ^1^	Continuous	Count	Number of episodes the drinking animal was disturbed by other animals or the corresponding behavior against other animals by the drinking animal. The sum of involvements in agonistic interactions of the cow at the water trough. Including displacement, head bump, pushing with core body [1].
Agonistic behaviors by the drinking cow	Continuous	Count	Disturbance of other animals by the drinking animal. Including displacement, head bump, pushing with core body.
**Agonistic behavior combined with other motions**			
Responding ^2^	Continuous	Count	Number of episodes of a cow responded in form of “ignoring, staring, moderate pushing, pushing aside, or fighting” due to agonistic behavior of another cow.
Ignoring	Continuous	Count	Number of episodes of a cow not showing any reaction due to agonistic behavior of another cow.
Staring ^1^	Continuous	Count	Two cows having direct eye contact, while at least one of them is visiting the trough.
Moderate push ^2^	Continuous	Count	Number of episodes of a cow slightly touched another one in the observation zone.
Pushing aside	Continuous	Count	Number of episodes of a cow pushing away another cow with the whole body or with the head inside the observation zone
Fighting	Continuous	Count	Number of episodes of a cow applying pressure head-to-head with another cow in the observation zone
Interruptions	Continuous	Count	Interruption of a drinking episode resulting from agonistic behavior.
Leaving	Continuous	Count	Termination of a drinking episode resulting from agonistic behavior.
Being groomed	Continuous	Count	Number of episodes a cow accepted licked by another cow inside the observation zone
Actively grooming others	Continuous	Count	Number of episodes a cow licked another cow inside the observation zone

^1^ partly based on Burkhardt et al. [1] and Burkhardt et al. [16] ^2^ based on Rousing and Wemelsfelder [39]. ^a^ based on Axegärd [40].

### 2.5. On-Farm Water Trough Evaluation

Water temperature and electrical conductivity were measured daily before and after the trials according to DIN EN 27888 (11/1993) [41] using a conductivity meter (EC-Meter G 1409, GHM GROUP-Greisinger | GHM Messtechnik GmbH, Regenstauf, Germany) and pH was measured according to DIN EN ISO 10523 (04/2012) [42] using a pH meter (COMBO Tester for pH/EC/TDS/C°, Hanna Instruments Deutschland GmbH, Voehringen, Germany) by dipping the instruments into the water at a depth of 2–5 cm (Supplementary Table S1).

### 2.6. Environmental Measures

Climate data were recorded to determine climatic changes during the experimental period. The weather loggers (KLIMALOGG PRO 30.3039, TFA Dostmann GmbH & Co. KG, Wertheim, Germany, temperature range outdoor −39.9 to +59.9 °C, sensitivity: 0.1 °C resolution; humidity range 1% to 99% RH, sensitivity 0.1% RH resolution) were positioned above the drinking trough at a height of 2 m (Figure 1). Ambient temperature and relative humidity (RH) were measured every 10 min. Daily mean and maximum ambient temperature and RH over the trial period are summarized in Supplementary Table S2.

### 2.7. Statistical Analysis

For the analysis, recorded behavior variables (*n* = 33 behavioral variables) were each summed and averaged for each individual cow. The obtained data of *n* = 42 cows were analyzed using R version 4.1.1.

A descriptive analysis of dairy cow drinking behavior (mean ± standard deviation, median, minima, maxima, range, percent range, frequencies) was conducted using the “calculate_stats” function. The range percent was calculated by dividing the range of values (range_val) by the maximum (max_val) and multiplying the result by 100. This results in a percentage representation of the range relative to the maximum of the data.

The normality of continuous variables was checked using a Shapiro–Wilk Test via the “dplyr” package. Normal distribution was only achieved by cube root transformation for 12 of 31 parameters. This, together with the small sample size, led to the decision to present and interpret the results only descriptively. However, as “agonistic behaviors” were normally distributed, this parameter was statistically analyzed to validate the index of competitive success.

Kendall rank correlations were conducted in R using the “ggcorrplot” package to determine the correlative relationship between body and performance traits and drinking behavior variables. All variables, including non-normal distributed variables, were analyzed using frequency distributions visualized by violin plots and scatter plots via the “ggplot2” package in R. In SAS, differences between normal distributed data were calculated using a linear mixed effect model with “competitive success” (low, medium, high) as a fixed factor.

To investigate the effect of “milk production” (categorized as two levels, low yielding cows: <30 L and high yielding cows: >30 L of milk per cow per day) on drinking behavior and agonistic interactions at the trough, a *t*-test was employed in R. Further, normality was only confirmed for the “Total duration of drinking”, the “Water volume consumed” and “Total number of agonistic behaviors”.

In all cases, *p* < 0.05 indicated a significant difference, whereas *p* < 0.01 was considered highly significant, and *p* < 0.10 was considered a tendency.

## 3. Results

### 3.1. Drinking-Related Behavior in Individual Dairy Cows

After milking, cows visited numerically most frequently the brush (mean: 6.4 ± 5.4 visits in 22 milkings, 29%), followed by immediately leaving the observation zone (mean: 6.01 ± 4.0 number of times in 22 milkings, 27.3%), drinking after another activity (mean: 5.6 ± 4.8 episodes in 22 milkings, 25.4%), visiting the water trough (16.0%, mean: 4.8 ± 3.9; visits in 22 milkings, 21.8%), eating concentrate (mean: 2.7 ± 4.1 times in 22 milkings, 12.3%), and most seldomly eating PMR (mean: 0.9 ± 1.1 times in 22 milkings, 4.1%) (Supplementary Figure S2). Five cows (11.9%) never drank directly after milking.

Visualization of individual drinking patterns within the herd demonstrates the highest numerical differences between cows in the duration of water intake (mean: 180.8 ± 195.3 s, range: 794.3 s), followed by the duration of drinking breaks (mean: 64.9 ± 61.5 s, range: 327.9 s) and number of drinking breaks (mean: 18.2 ± 47.3, range: 285 breaks), the total duration of drinking (78.4 ± 47.5 s, range: 223.7 s), the number of water intake periods (mean: 32.1 ± 48.5, range: 287 periods) and the number of sips (mean: 52.5 ± 31.9, range: 110.7 sips). From 26 (56%) cows drinking shorter than 78 sec, only one cow was interrupted more than two times. The mean volume consumed per sip was 0.2 ± 0.1 L (range: 0.4 L) (Supplementary Figure S2), and the mean water volume consumed per cow during the trial period ranged from min: 1.2 L (in case of trough visit with water intake) to max: 24.2 L (mean: 7.9 ± 4.6 L). Of all cows, 23.8% (10/42 cows) drank on average less than 5 L per milking at the trough next to the milking parlor, and 14.3% (6/42 cows) drank shorter than the average of 30 s.

Except for two cows, all animals were involved in agonistic interactions (*n* = 232 direct physical interactions, *n* = 164 indirect, non-physical interactions (e.g., “staring” (*n* = 140) and “ignoring” (*n* = 25)) during the study period, but only one-third of the animals initiated these. One of the cows that was never involved in agonistic interactions never drank immediately after milking. The most prevalent agonistic interactions recorded were “staring” (35.3%), “pushing aside” (34.5%), or “moderate pushing” (19.9%). Most seldomly, “ignoring” (6.3%) and “fighting” (4.0%) were observed (Supplementary Figure S3). In total, 72% of the cows were interrupted during drinking, and 83% left the observation zone at least once due to agonistic behaviors. Of *n* = 92 interruptions, 23 involved “staring” (25.0%) as one agonistic behavior, and 7 were interruptions due to “staring” only (7.6%).

### 3.2. Association of Individual Body and Performance Traits and Drinking-Related Behavior in Dairy Cows

#### 3.2.1. Body and Performance Traits and Their Influence on Drinking Behavior

Milk production-related traits and body weight varied more than other body-related traits among the experimental cows (Supplementary Figure S1).

**Figure 2:**
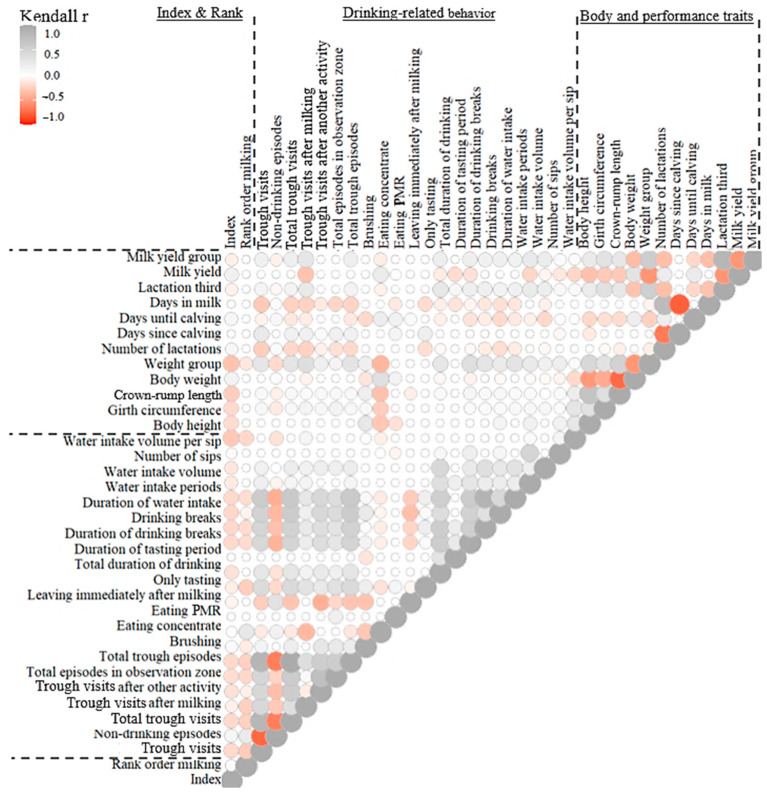
Kendall correlations (*p* ≤ 0.05) between drinking-related behavior characteristics and body and performance traits of the experimental dairy herd. The circle size indicates the level of correlation.

Milk yield and the number of lactations correlated positively with the number of trough visits, the total duration of drinking, the duration of water intake and drinking breaks, and the water volume consumed (Figure 2). All of the ten highest yielding cows drank at least eight times at the trough next to the milking parlor, tasted the water at least 4 s, and never left the trough after tasting only. They had a mean consumption of at least 7 L, with at least 21 sips in more than 37 s of drinking duration.

Categorizing the herd in two groups of *n* = 21 cows each, low yielding (<30 L milk per day) and high yielding (>30 L milk per day) showed that high yielding cows consumed twice as much water (high yielding cows 9.79 ± 0.9 L; low yielding cows: 5.8 ± 1.0 L), twice as long (high yielding cows 100.1 ± 9.2 s; low yielding cows: 55.7 ± 9.2 s), and were twice as often involved in agonistic interactions at the trough (high yielding cows 6.5 ± 1.0 times; low yielding cows: 3.5 ± 1.0 times) (*p* < 0.05).

#### 3.2.2. Body and Performance Traits of the Experimental Herd and Its Influence on Agonistic Interactions

**Figure 3:**
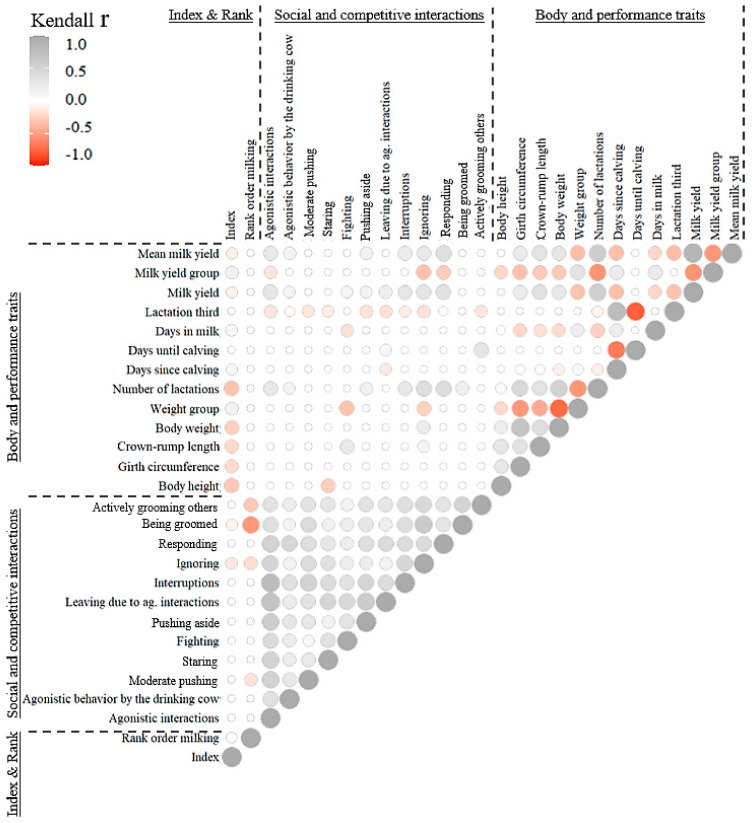
Kendall correlations (*p* ≤ 0.05) between social and competitive interaction-related characteristics and body and performance traits of the experimental dairy herd. The circle size indicates the level of correlation.

Solely ignoring correlated positively with the animal’s body weight (r = 0.2; *p* < 0.05). The higher the milk yield, respectively, the number of lactations, the higher the involvement in agonistic interactions (r = 0.1; r = 0.3), ignoring (r = 0.3; r = 0.4), responding (r = 0.3; r = 0.4), and pushing aside (r = 0.1, r = 0.2) (*p* < 0.05). Days in milk, days until or since calving, or girth circumference only had minor or no effects on agonistic interactions (Figure 3). The fourth highest-yielding cow started most often agonistic interactions while drinking (14 times in 22 milkings).

### 3.3. Dairy Cows Drinking Behavior under Consideration of Competitive Success at the Trough

#### 3.3.1. Association of Competitive Success and Body and Performance Traits

In total, 42 lactating cows were classified into three groups (“low success”, “medium or no competitive interactions”, and “high success”) following the index of competitive success by McDonald et al. [28]. Most of the cows were grouped in “medium success or no competitive interactions” (59.5%; *n* = 25), followed by “low competitive success” (21.4%; *n* = 9) and “high competitive success” (19.0%; *n* = 8) at the trough. Cows with high success at the trough had numerically more days in milk but a lower milk yield, body height, body girth, crown–rump length, and body weight compared to cows with low competitive success at the trough (Table 1).

#### 3.3.2. Effects of Competitive Success on Drinking Behavior

**Figure 4:**
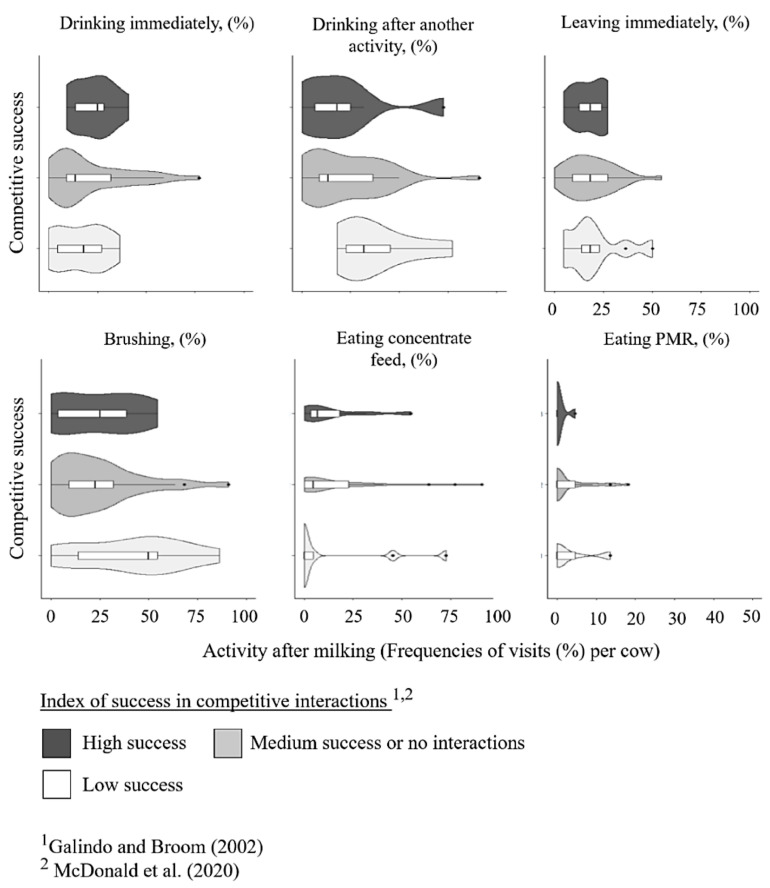
The violin plots show the distribution of different activities after milking (frequencies of visits in 22 milkings per cow) across dominance groups (high success, medium success, low success) where width represents data density, wider sections indicate higher concentrations, and median and interquartile range are marked [28,37].

**Figure 5:**
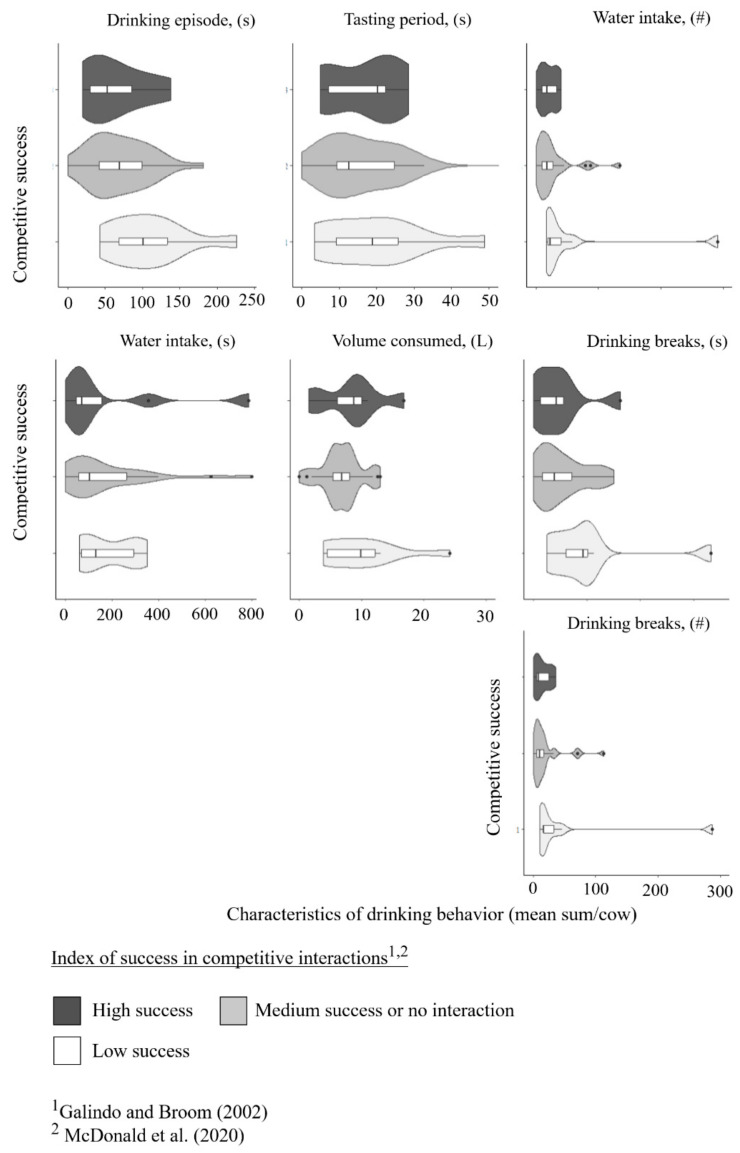
Distribution of different drinking behavior parameters shown by violin plots (mean sum per cow in 22 milkings) of three dominance groups (high success, medium success, low success). Width represents data density, wider sections indicate higher frequencies, and median and interquartile range are marked [28,37].

Including cows that returned from another activity directly after milking to drink, the group of cows with high success at the trough had numerically fewer trough visits (high success: mean: 8.6 ± 6.1 episodes; low success: 12.0 ± 6.3 episodes in 22 milkings), and fewer trough visits immediately after milking (high success: mean: 3.6 ± 1.8 episodes; low success mean: 4.8 ± 2.8 episodes in 22 milkings), but cows with a low competitive success drank numerically more frequently after another activity (high success: mean: 5.0 ± 5.1 episodes, low success: mean: 7.2 ± 3.0 episodes in 22 milkings) and had numerically more total episodes in the observation zone (high success: mean: 23.2 ± 6.9 episodes; low success: mean: 27.2 ± 2.5 episodes in 22 milkings) (Figure 4). The water consumed at the trough seems to decrease with competitive success, as the group of high success cows had lower water volumes consumed than medium and low success cows having the highest water volume consumed (high success: mean: 7.0 ± 3.9; median success or no competitive interactions: mean: 7.9 ± 4.8; low success: mean: 9.5 ± 4.9 L per cow), while cows with high success had on average a more than 30 s shorter duration of drinking (high success: mean: 63.3 ± 31.9; low success: mean: 94.6 ± 33.2 periods in 22 milkings). From the referred twenty-six cows drinking shorter than 78 s, only two cows belonged to the group of low competitive success: one of them was the only cow that was interrupted more than two times due to agonistic behaviors. Concerning the water intake periods and drinking breaks, cows with low competitive success seemed to have comparatively many water intake periods (high success: mean: 16.1 ± 9.8, max: 32; low success: mean: 36 ± 24.9 max: 79 periods in 22 milkings), respectively, drinking breaks (high success: mean: 10.4 ± 8.0, max: 27; low success: mean: 27 ± 25.1, max: 71 breaks in 22 milkings) (Figure 5).

Two of the nine cows with low competitive success had drinking breaks on average longer than 100 s (all cows mean 25.4 ± 47.3 s) and two of the cows with high competitive success had water intake periods longer than on average 290 s (~5 min) (all cows mean: 180.8 ± 195.5 s).

#### 3.3.3. Association of Competitive Success at the Trough and Agonistic Behavior of Dairy Cows

The number of involvements in agonistic behavior in general differed significantly between groups of competitive success at the trough (high success: mean: 4.3 ± 3.5, medium success: mean 5.4 ± 5.6; low success: mean: 3.8 ± 1.3 involvements in 22 milkings) (*p* < 0.05). However, cows having highly competitive success at the trough twice as often started competitive interactions compared to cows with low success (high success: mean: 3.1 ± 1.5 times; low success: mean: 1.7 ± 1.1 times in 22 milkings). Likewise, receivers of agonistic interactions were most often cows with low competitive success and seldomly cows with high success (high success: mean: 0.5 ± 0.5 times; low success: mean: 6.7 ± 3.4 times in 22 milkings). Cows with low competitive success were at least three times the receiver of agonistic interactions. Interruptions were numerically lower for cows in the group of high success (high success: mean: 1.4 ± 1.1 times; low success: mean: 2.0 ± 1.2 times in 22 milkings).

In total, cows with high competitive success reacted numerically twice as often (by “ignoring”, “staring”, “moderate pushing”, “pushing aside”, or “fighting”) than cows with low competitive success (high success: mean: 1.3 ± 1.5 times; low success: mean: 0.4 ± 0.9, times in 22 milkings). For both groups, non-physical interactions such as “staring” appeared more frequently (high success: mean: 3.4 ± 2.7 times; low success: mean: 3.6 ± 3.9 times in 22 milkings) than direct physical contacts as “moderate pushing” (high success: mean: 1.5 ± 2.8; low success: mean: 1.6 ± 1.7 times in 22 milkings), “pushing aside” (high success: mean: 2.6 ± 0.7 times; low success: mean: 2.8 ± 2.3 times in 22 milkings), or “fighting” (high success: mean: 0.4 ± 0.7 times; low success: mean: 0.0 ± 0.0 times in 22 milkings).

## 4. Discussion

Our study describes dairy cows’ drinking behavior at an individual animal level, considering performance traits and competitive success. It thereby includes a precise description of agonistic interactions and behavior exiting the milking parlor and facing the water trough, using a total of *n* = 33 behavioral variables.

Andersson [35] and Cardot et al. [26] indicated in their studies that dairy cows meet most of their daily water demand after milking. Despite the strong relation of milk production and water intake revealed by several studies, in the current study, cows first visited the brush or left the observation zone before drinking at the trough next to the milking parlor. Nevertheless, as the behavioral observation was limited to a specific observation zone, it is likely that cows left the area and drank somewhere else. Considering the relatively short distance to the next available trough in our study, we expected some effects to be more obvious with higher distances. This is particularly evident in the high variation of the amount a cow consumed, as the minimum recorded volume of 1.2 L does not meet the demand for water after milking, even at low milk yields. Authors indicate an average water intake of dairy cows comprising 10–12 L per drinking episode [4,26,43]. Furthermore, one-fifth of the herd drank on average less than 5 L, and five cows never drank at the trough directly after milking (two of the group with low success and three of the group with medium success or no interaction). Cows with higher competitive success seem to be more able to satisfy their water demand without interruption. Cows belonging to the group of cows with high or medium success at the trough drank, except for one cow, all shorter than 78 s (56% of all animals) and were less than two times interrupted by agonistic behavior.

The interaction of the inability to access water due to blocking, on the one hand, is amplified by the great variation in the water intake-related episodes of the individual animal, on the other hand. Animals that need longer to consume the same amount of water are potentially additionally discriminated against. This is also reflected in the ratio of drinking duration and water volume consumed (all cows: 0.11 L/s, high success: 0.12 L/s, medium success 0.11 L/s, low success 0.09 L/s). However, the low ratio of drinking duration and water volume consumed in cows with low competitive success may also be due to a preference by these animals to drink at a different drinking trough than the one closest to the milking parlor. Under practical conditions, the frequency of sips taken during a drinking episode might serve as an indicator to estimate water intake on an individual basis and thus may assist in identifying potentially discriminated animals, as it correlated positively with the water consumed, as suggested by Burkhardt et al. [16].

Several authors describe the high prevalence of agonistic interactions at drinking troughs [28,35,43]. This is confirmed by our study, as almost all cows were involved in agonistic interactions at the trough, while only one-third of the animals started these. The level of detail in describing agonistic interactions considerably varies among studies. Mostly, the definition of agonistic interactions includes displacements that provoke withdrawals of body parts (body pushing, head butting, head pressing, body sniffing [44]) but exclude non-physical interactions for technical reasons [43,45,46]. Our study found that non-physical, indirect interactions account for almost half of all agonistic interactions (41.0%), with seven non-physical interactions (“starring” only) leading to interruptions of drinking events. Nevertheless, direct interaction led to more displacements at the trough overall. Several authors indicate that direct physical contacts (butting, pushing) alone are insufficient to reflect agonistic behavior within the herd, as subtle interactions and mutual awareness already influence movements and behaviors and thus, potentially limit the access to the water trough [47,48,49]. Based on the findings of this and other studies, non-direct agonistic interactions should be included in assessing dairy cows’ behavior, and it is crucial that dairy farmers are aware of impairments by competition that are hardly visible.

We hypothesized that differences in physiological and body characteristics lead to variations in the drinking behavior among individuals. The effects of parity on drinking behavior and water consumption are hardly investigated. Following Dado and Allen [50], multiparous cows drank more (89.5 L/d) than primiparous cows (63.2 L/d). However, this is likely due to higher milk production.

The current study indicates that the higher factors known to significantly affect water consumption, such as milk production-related traits [19,40], the higher the number of trough visits, the duration of drinking and of water intake, drinking breaks, and the water intake volume. Several studies indicate a positive correlation between milk yield and trough visits (r = 0.37) [26], respectively, water intake (r = 0.4; r= 0.5, *p* < 0.01) [22,26,51]. Moran [52] suggests for each kg of milk an additional water provision of 6–7 L. Days in milk only correlated weakly with trough visits (r = 0.09; *p* < 0.01) [26]. Lactation number correlated positively with trough visits (r = 0.41) [26] and water intake [22].

The current study not only used extensive correlation analyses to identify associations between drinking behavior and milk production-specific traits but also observed higher yielding cows in contrast to lower yielding cows consuming twice as much water, in double the time, and interacted twice as often agonistically with others at the trough. Additionally, the milk yield, respectively, and the number of lactations correlated positively with the involvement in agonistic interactions, as well as a cow responding, pushing aside, or ignoring. One of the ten highest yielding cows started most often agonistic interactions while drinking (14 times in 22 milkings). The observed increase in agonistic behavior in cows with higher production traits raises the question of whether nutritional grouping based on milk yield or days in milk can counteract this, as it potentially leads to very high competition in high-yielding groups [53]. Nutritional grouping of dairy cows has been addressed in the past by some scientists regarding social dominance [54]. Nevertheless, water consumption was not considered, even though recommendations for the drinking space per cow are limited to 10 cm per animal, compared to 36–61 cm for the calculated feeding space [55]. This disproportion is justified by the permanent availability of water in livestock barns, but the fact that a large number of animals feel simultaneous thirst after milking [14], especially in parlor milking systems, is neglected.

Foris et al. [43] mentioned “that long bouts were not caused by a technical error but rather by cows truly occupying the bin for a long time”. Similarly, Burkhardt et al. [56] found cows to guard water resources without drinking actively, especially during periods of higher ambient temperature. Considering that high yielding dairy cows require more water [22,26,51], compete more for access to water troughs and have higher heat production [56] potentially leading to longer periods at water troughs to cool [16], it seems necessary to move away from general to yield adjusted recommendations or milking parlors equipped with water troughs.

In order to investigate the influence of social hierarchy in dairy cows, the current study adapted a dominance index implemented by Galindo and Broom [37] to determine the social status of the individuals within a herd, which was later adapted in several studies [28,31]. Ratios of cows having low, medium, and high competitive success in the current study were consistent with the findings of McDonald et al. [28]. In our study, the parameter “agonistic behaviors” was statistically analyzed to validate the index of competitive success. Ensuring each cow’s involvement in at least eight agonistic interactions would have required an extension of the trial period, which was not feasible.

Following the findings of Hohenbrink and Meinecke-Tillmann [31] and Axegärd [40], neither age nor the lactation period significantly affected social dominance. Nevertheless, cows with high success at the trough appeared to have a higher mean in days in milk but a lower median in milk yield, body height, body girth, crown–rump length, and body weight. This contrasts with Andersson [35], who observed a significant relationship between rank and body weight. Similarly, following Hohenbrink and Meinecke-Tillmann [31], body condition score is positively correlated with the dominance index. Sołtysiak and Nogalski [45] describe a higher milk yield and body weight in higher-ranked cows. In their study, all cows had a similar age, while in our experiment, the individuals of the experimental herd ranged between one and seven lactations and thus did body-related traits. Moreover, a proportion of cows was recently reintegrated after calving, and, therefore, social hierarchy may have been subject to changes more than in other studies.

In terms of the competitive success at the trough in association with the drinking behavior, we expected that cows with a high competitive success predominantly use the trough next to the milking parlor directly after milking, and those cows consume more water, drink longer, and show more extended periods of water intake. The hypothesis that cows with a high competitive success predominantly use the trough next to the milking parlor directly after milking was not confirmed; however, cows with low competitive success drank more often after pursuing another activity first, possibly indicating a waiting for free trough access. This would be in accordance with McDonald et al. [28], who reported in a study about dairy cows’ social behavior at the trough under hot weather conditions that cows with low competitive success shift their drinking times to avoid competition. Nevertheless, the frequency of drinking was not affected by the competitive success of the individuals. This contrasts the findings of Andersson [35], who found that low-ranking cows drink earlier than high-ranking ones. Coimbra et al. [5] investigated cows’ drinking behavior in a rotational grazing system in different trough locations (in a corridor vs. in a paddock) under different shade availabilities. Placing the trough in a corridor resulted in more and longer trough visits of dominant cows compared to subordinates. This is partly in accordance with a behavioral study of Axegärd [40], who reported a shorter duration in drinking but fewer trough visits of low-ranked cows. In our findings, cows with high success had numerically fewer and, on average, 30 s shorter trough visits and drank less than cows with low success, but also numerically fewer interruptions due to agonistic behaviors. McDonald et al. [28] did not find any effect of competitive success on the time cows spent at the trough, but water consumption increased with increasing levels of competitive success. This contrasts the findings of Andersson [35] and our findings that water consumption decreased with competitive success. Nevertheless, our study only represents a limited time sequence of the day. As the total number of trough visits includes cows that return, it can be assumed that cows of the group with low competitive success needed several attempts to fulfil their demand and were more frequently interrupted by agonistic interactions. This could also explain the numerically higher total episodes observed in the observation zone, including brushing, eating PMR, or concentrate and thereby possibly waiting until access to the trough is free in the group with low competitive success. Andersson [35] likewise observed some cows to be anxious to drink and rather wait than drink with others at the same time. Our hypothesis that cows with high competitive success would start more frequently agonistic interactions than cows with less competitive success at the trough was confirmed, as the group with high competitive success was numerically more frequently the aggressor and reacted more frequently on agonistic interactions. Differences in the studies outcomes may be due to both the composition of the experimental herds in age, milk yield and feeding regime, as well as the different definitions of ranking (index of competitive success vs. the use of a master chart described by Schein and Fohrman [57], making harmonization of the experimental conditions highly desirable.

## 5. Conclusions

The heterogeneity of the herd in terms of body and performance traits was reflected in the drinking behavior of the individuals, which varied considerably. Especially with increasing milk yield, water consumption-related behaviors increased. Moreover, social competition is largely characterized by non-physical interactions. Agonistic behavior at the trough started predominantly from cows with high competitive success. Cows with low competitive success seem to wait to access water and pursue other activities in the meantime to avoid contact with others at the trough and are also interrupted more frequently. Therefore, cows with low competitive success most likely need several attempts to fulfill their water requirements.

In summary, this study shows the complexity but also the potential of including drinking behavior for evaluating water management on dairy farms and highlights the need for reviewing current recommendations for water supply to take physiological needs and differences into account. Based on our results, of particular importance is an increase in the number of drinking troughs in high-performance groups. Further studies might address potential beneficial effects of providing water during milking and thus guaranteeing free water access for all animals during times of high water loss due to milk excretion. Not only might subordinate cows benefit physiologically and welfare-based by free water access at least twice daily during milking, but aggressive behaviors at the trough might also be reduced.

## Figures and Tables

**Table 1:** Characteristics of the experimental lactating dairy cow herd, continuously held in a free-range barn in July 2022, considering rank groups defined as success in competitive interactions (high success, medium success or no interaction, low success). Body and performance traits are shown as median.

Variable	All Cows					Competitive Success		
						High (*n* = 8)	Medium (*n* = 25)	Low (*n* = 9)
	Mean ± SD	Median	Min	Max	Range (%) *	Mean ± SD	Mean ± SD	Mean ± SD
Days in milk (d)	367.4 ± 69.5	360.0	268.0	572.0	304 (53.1)	352.0 ± 40.5	381.0 ± 77.2	325.5 ± 39.0
Lactation number	2.3 ± 1.5	2.0	1.0	7.0	6.0 (85.7)	2.3 ± 2.0	2.3 ± 1.2	3.8 ± 1.5
Milk yield (kg/d)	30.4 ± 7.5	29.8	11.5	43.9	32.4 (73.9)	30.0 ± 5.7	30.3 ± 8.5	32.2 ± 4.3
Body height (cm)	149.8 ± 3.7	149.5	144.0	158.0	14.0 (8.8)	147.9 ± 2.3	149.9 ± 3.8	152.3 ± 3.3
Girth circumference (cm)	207.1 ± 9.5	205.0	193.0	227.0	34.0 (14.9)	203.9 ± 5.8	206.4 ± 9.3	214.5 ± 11.9
Crown–rump length (cm)	215.8 ± 7.0	216.5	204.0	229.0	25.0 (10.9)	215.5 ± 7.2	214.8 ± 7.1	221.5 ± 3.5
Body weight (kg)	670.0 ± 75.6	644.0	516.0	858.0	342.0 (39.8)	640.0 ± 51.6	667.1 ± 76.7	723.7 ± 80.4
Days until calving (d)	248.8 ± 108.8	284.0	13.0	394.0	381.0 (96.7)	220.3 ± 67.5	268.5 ± 112.0	196.8 ± 123.9
Days since calving (d)	172.5 ± 112.7	165.5	0.0	499.0	499.0 (100)	202.9 ± 58.1	165.1 ± 125.5	169.2 ± 110.8

* Percentage of the range relative to the maximum of the data for comparative visualization of the range of body characteristics between individuals (calculated by dividing the range of values by the maximum and multiplying the result by 100).

## Data Availability

The data presented in this study are available on request from the corresponding author. The data are not publicly available due to further scientific use to establish AI.

## Supplementary Materials

The following supporting information can be downloaded at: https://www.mdpi.com/article/10.3390/ani15040534/s1, Table S1: Physico-chemical livestock drinking water quality variables offered water. Table S2: Average and maximum daily values considering time of the day (morning- and evening trials) for water temperature, ambient temperature and relative humidity (RH). Figure S1: Average and maximum daily values considering time of the day (morning- and evening milking) for water temperature, and ambient temperature. Figure S2: Dairy cows individual body- and performance traits sorted ascending by count; Dairy cows individual drinking behavior within the herd, illustrated by six behavioral variables sorted ascending by size, animals are encoded by their individual ear tag identification number. Figure S3: Dairy cows expressions of agonistic behavior at the trough, illustrated by six behavioral variables sorted ascending by size, animals are encoded by their individual ear tag identification number. References [34,58,59] are cited in the supplementary materials.

## Abbreviations

BORIS, Behavioral Observation Research Interactive Software; PMR, Partly mixed ration.
