# Supplementary material for: Association Between Individual Animal Traits, Competitive Success and Drinking Behavior in Dairy Cows After Milking
Source: Animals (Basel). 2025 Feb 13;15(4):534. doi: 10.3390/ani15040534 (PMC11852067; doi:10.3390/ani15040534)
Supplement: Supplementary file 1 [file animals-15-00534-s001.zip › animals-3461291-supplementary.pdf]

<u>**Supplementary file**</u>

Association Between Individual Animal Traits, Competitive Success and Drinking Behavior in Dairy Cows After Milking

Franziska Katharina Burkhardt, Rieke Wahlen, Jason Jeremia Hayer and Julia Steinhoff-Wagner

Supplementary Table S1. Physico-chemical livestock drinking water quality variables offered water.

Variable	Value	Reference value
<u>Water from the local municipal utility[a]</u>		
Salinity (mg/L)	200.0	< 25.000[b]
Calcium (mg/L)	93.2	< 500[b]
Nitrate (mg/L)	50.0	< 300[b]
Sulfate (mg/L)	250.0	< 500[b]
Kalium (mg/L)	3.2	-
Mangan (mg/L)	0.05	< 1000[c]
Iron (mg/L)	0.2	< 3[b]
<u>Daily trough water measurements</u>		
pH value	7.6 (7.23 7.8)	5–9[b]
Electrical conductivity (µS/cm)	719.7 (229.0 807.0)	< 3000[b]

[a] Based on Freisinger Stadtwerke [34]

[b] Based on Kamphues et al. [58]

[c] Based on Olkowski [59].

Supplementary Table S2: Average and maximum daily values considering time of the day (morning- and evening trials) for water temperature, ambient temperature and relative humidity (RH).

	Morning		Evening	
Variable	Mean	SE	Mean	SE
Mean				
Water Temperature (°C)	15.8	0.4	18.2	0.5
Ambient Temperature (°C)	20.0	0.7	20.2	0.8
RH (%)	63.3	1.8	63.7	1.8
Maximum				
Water Temperature (°C)	20.5	-	20.5	-
Ambient Temperature (°C)	24.4	1.0	24.6	1.1
RH (%)	77.5	1.8	77.7	1.8

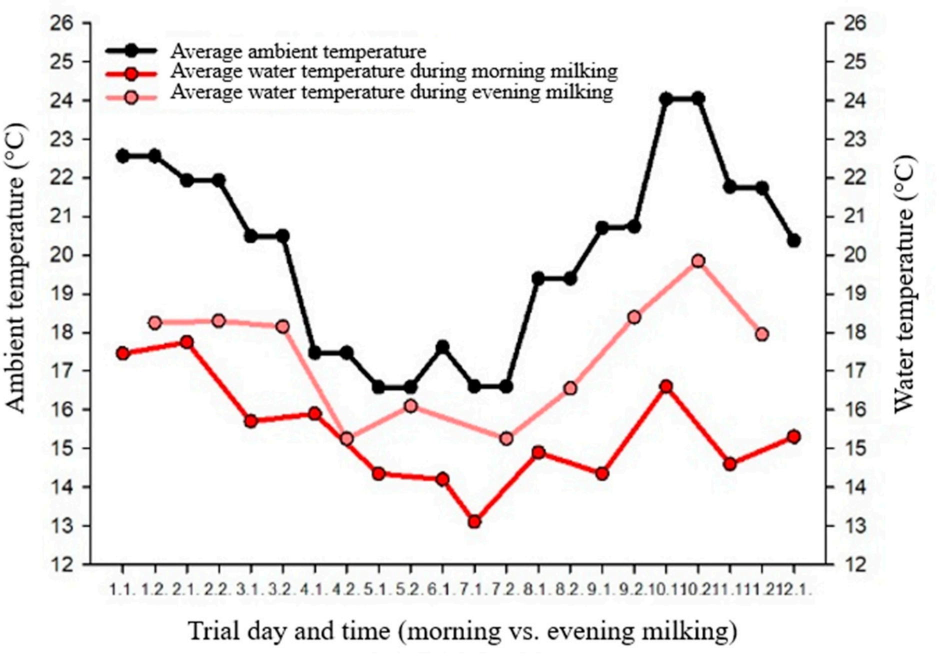

Supplementary Figure S1: Average and maximum daily values considering time of the day (morning- and evening milking) for water temperature, and ambient temperature.

Body- and performance traits of the experimental herd

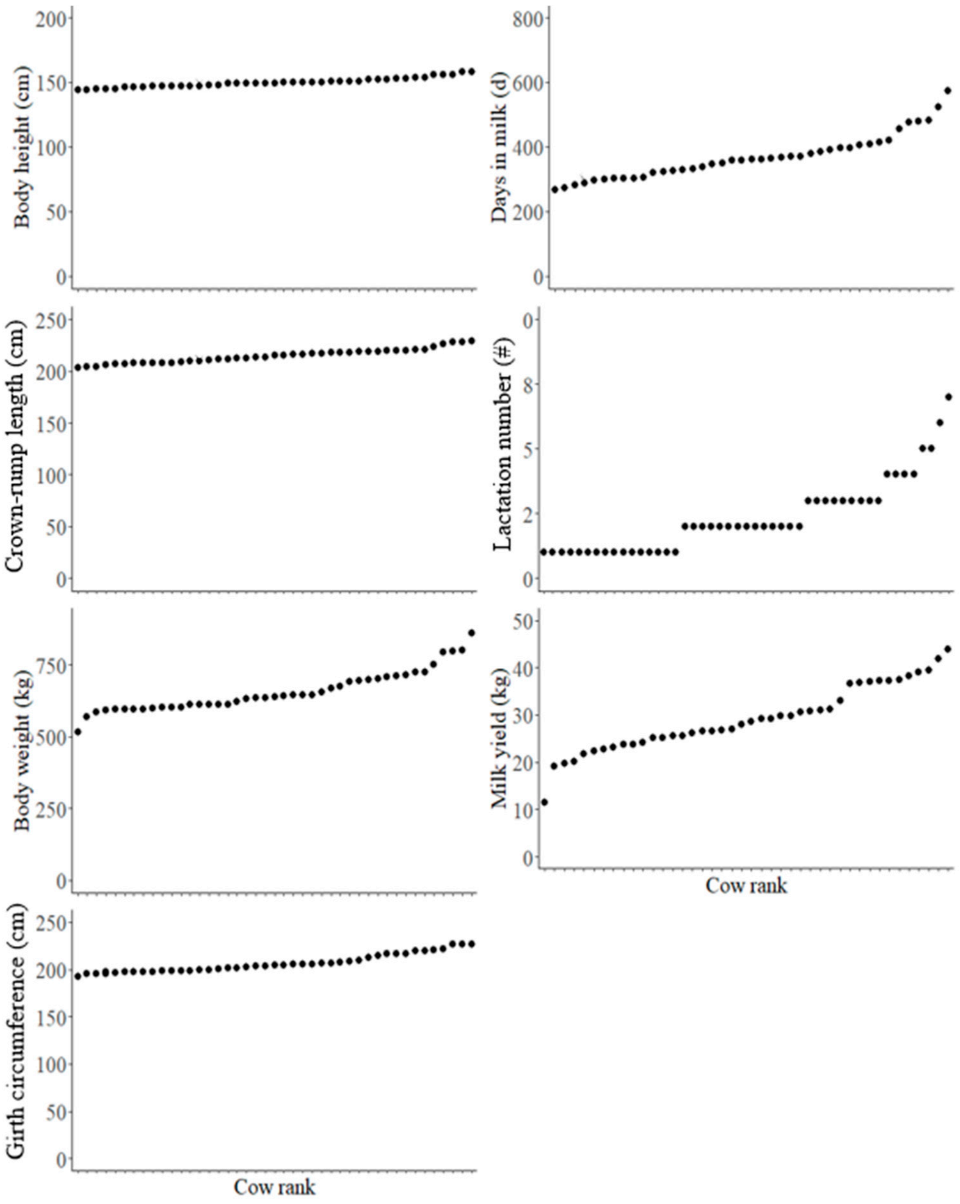

Supplementary Figure S2: A. Dairy cows individual body- and performance traits sorted ascending by count.

Activities after milking (#/cow)

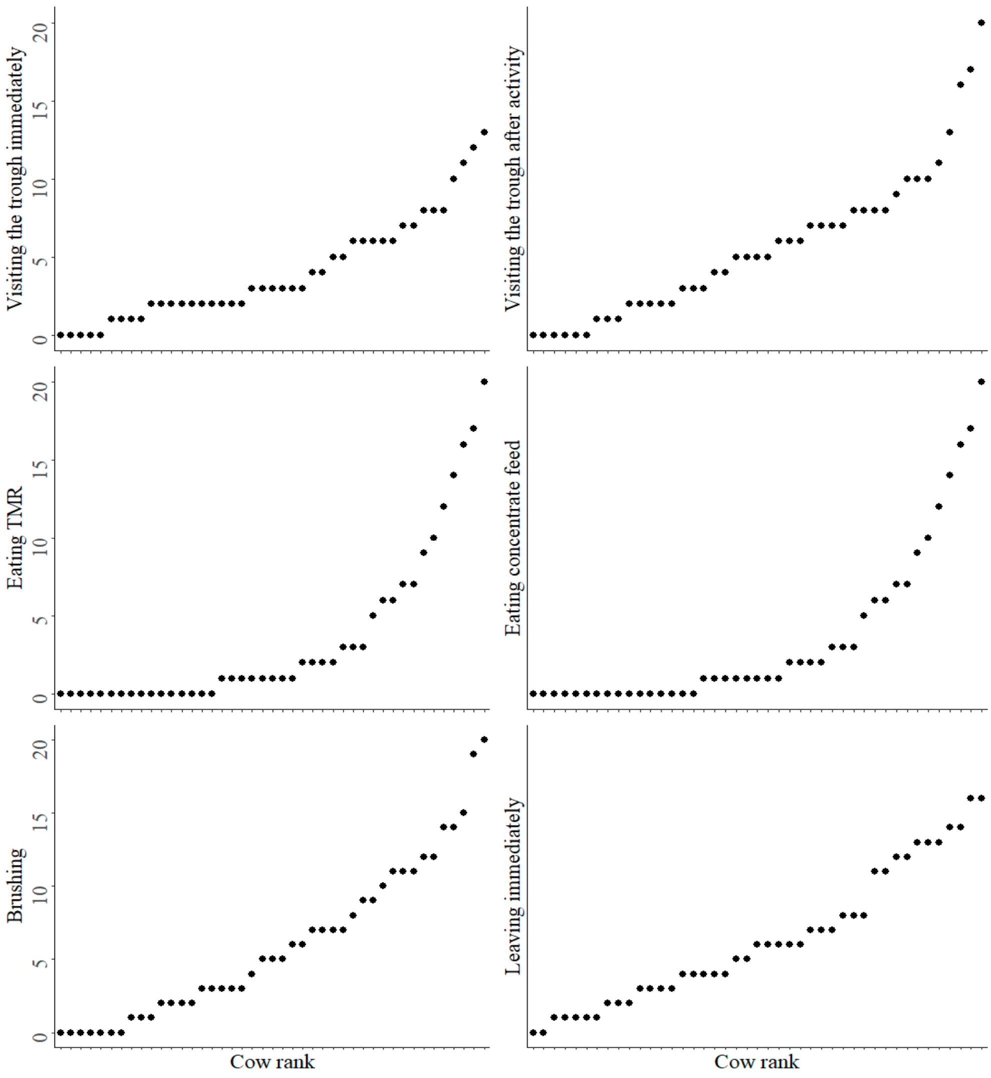

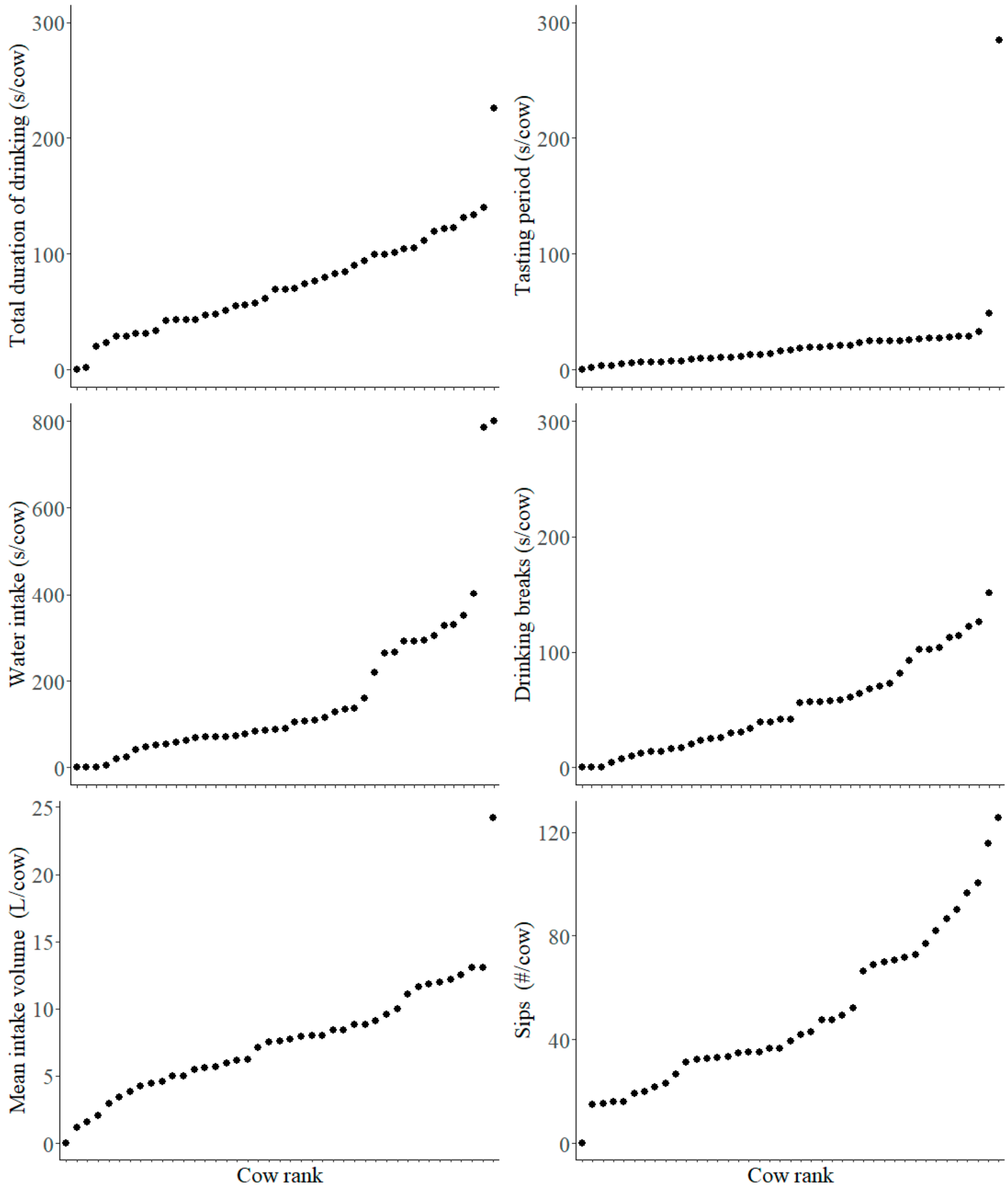

Supplementary Figure S2: B. Dairy cows individual drinking behavior within the herd, illustrated by six behavioral variables sorted ascending by size, animals are encoded by their individual ear tag identification number.

Type of agonistic interactions (#/cow)

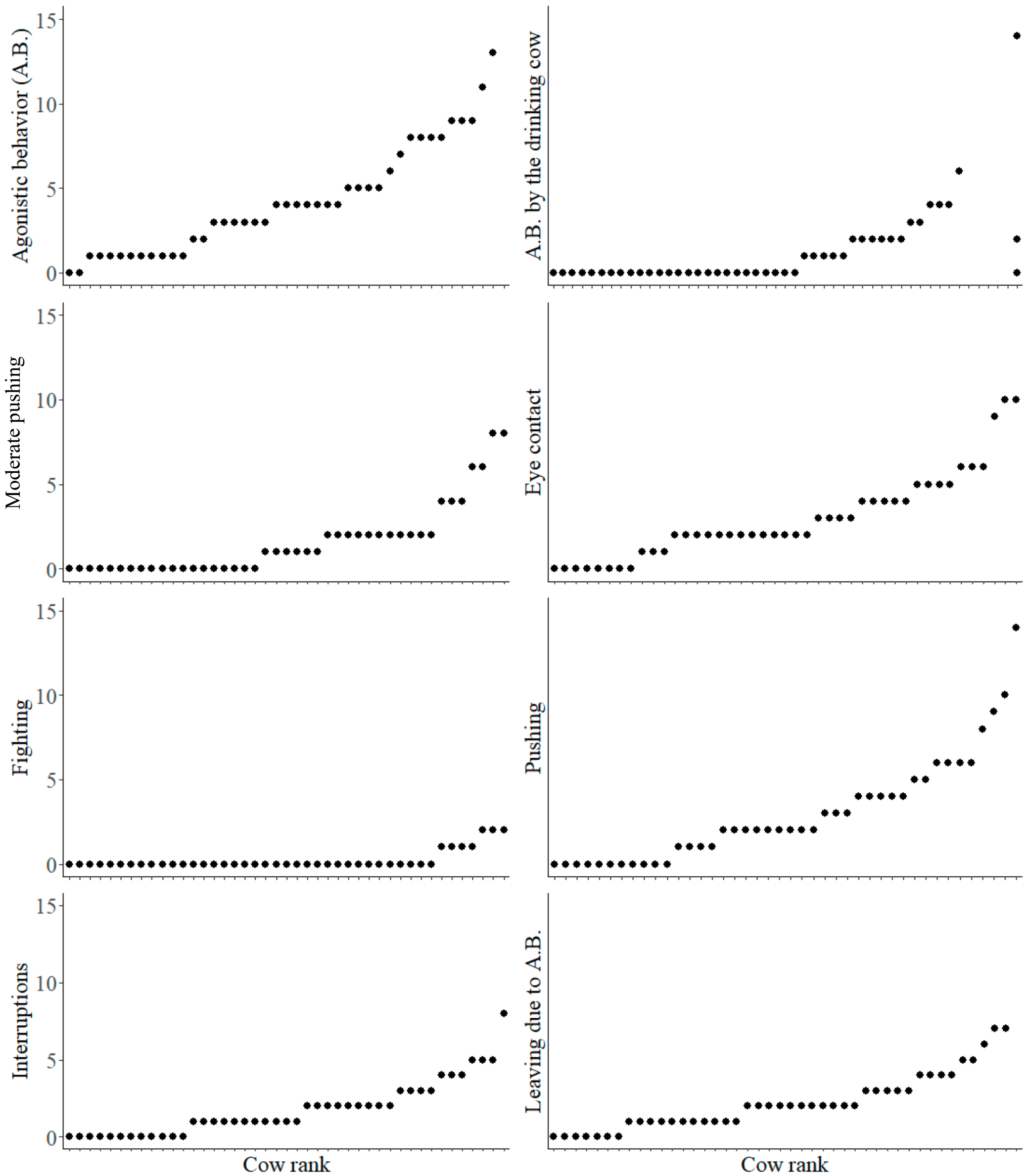

Supplementary Figure S3: Dairy cows expressions of agonistic behavior at the trough, illustrated by six behavioral variables sorted ascending by size, animals are encoded by their individual ear tag identification number
